# Association of the DNA Methylation of Obesity-Related Genes with the Dietary Nutrient Intake in Children

**DOI:** 10.3390/nu15132840

**Published:** 2023-06-22

**Authors:** Priyadarshni Patel, Vaithinathan Selvaraju, Jeganathan Ramesh Babu, Thangiah Geetha

**Affiliations:** 1 Department of Nutritional Sciences, Auburn University, Auburn, AL 36849, USA; 2 Boshell Metabolic Diseases and Diabetes Program, Auburn University, Auburn, AL 36849, USA

**Keywords:** childhood obesity, DNA methylation, epigenetics, health disparities, folate intake, methyl donors, dietary intake

## Abstract

The occurrence of obesity stems from both genetic and external influences. Despite thorough research and attempts to address it through various means such as dietary changes, physical activity, education, and medications, a lasting solution to this widespread problem remains elusive. Nutrients play a crucial role in various cellular processes, including the regulation of gene expression. One of the mechanisms by which nutrients can affect gene expression is through DNA methylation. This modification can alter the accessibility of DNA to transcription factors and other regulatory proteins, thereby influencing gene expression. Nutrients such as folate and vitamin B12 are involved in the one-carbon metabolism pathway, which provides the methyl groups necessary for DNA methylation. Studies have shown that the inadequate intake of these nutrients can lead to alterations in DNA methylation patterns. For this study, we aim to understand the differences in the association of the dietary intake between normal weight and overweight/obese children and between European American and African American children with the DNA methylation of the three genes *NRF1*, *FTO*, and *LEPR*. The research discovered a significant association between the nutritional intake of 6–10-years-old children, particularly the methyl donors present in their diet, and the methylation of the *NRF1*, *FTO*, and *LEPR* genes. Additionally, the study emphasizes the significance of considering health inequalities, particularly family income and maternal education, when investigating the epigenetic impact of methyl donors in diet and gene methylation.

## 1. Introduction

Childhood overweight and obesity results from consistently consuming more energy than needed and is influenced by a combination of genetics, lifestyle choices, the obesogenic environment, and social factors [1]. While there is evidence of a genetic component involved in childhood obesity, the significant rise in obesity rates among children cannot be solely attributed to genetic changes [2,3]. This suggests that interactions between genes and the environment are likely driving the epidemic of childhood obesity. Obesity, which is defined as the abnormal accumulation of excess body fat, can lead to various health issues such as blood lipid disorders, high blood pressure, insulin resistance, type 2 diabetes, metabolic syndrome, cardiovascular disease, and liver fat accumulation [4]. The prevalence of obesity is rapidly increasing in modern society, with an estimated 58% of adults worldwide expected to meet the criteria for obesity by 2030 [5]. The literature shows that approximately 55% of children who are obese will continue to be obese during their teenage years [6]. Roughly 80% of obese teenagers remain obese in adulthood, and approximately 70% stay obese beyond the age of 30 years [6]. When we look closer, obesity disproportionately affects racial minority groups, especially Hispanic and Black groups; therefore, it is important to understand the causes and reduce the prevalence of childhood obesity [7].

As the rise in childhood obesity rates in recent decades cannot be fully attributed to genetics alone, factors such as nutrition and lifestyle in our surroundings may also play a role in this trend. These factors can influence gene expression without altering the DNA sequence, a phenomenon known as epigenetics [8]. Epigenetics explores how external influences, such as lifestyle choices, exercise, toxin exposure, and diet, can impact gene expression [9,10,11]. These modifications play a role in various conditions, including obesity [12,13,14], type 2 diabetes [15], metabolic syndrome [16], insulin resistance [17], and cancer [18]. Conrad Waddington introduced the concept of epigenetics in 1942, which involves chemical modifications that affect how the body interprets DNA [19]. The most extensively researched epigenetic changes include DNA methylation, histone modifications, and non-coding RNAs [8]. Importantly, various therapeutic approaches, such as a low-calorie diet [20,21], bariatric surgery [22,23], and physical activity [24,25], can reverse these epigenetic markers that occur during obesity management. Nutrients can also serve as a source of epigenetic modifications and reverse specific disease-associated epigenetic markers [26,27]. As a result, nutritional epigenetics has emerged as a novel mechanism that explains how nutrition interacts with genes, providing evidence of its role in metabolic diseases.

Early-life nutrition causes long-term alterations in DNA methylation, which have adverse effects on individual health. Nutrients can exert their influence by directly inhibiting epigenetic enzymes such as DNA methyl transferase (DNMT), Histone deacetylase (HDAC), or Histone acetyltransferases (HAT) or by changing the availability of the substances required for those enzymatic reactions. As a result, the expression of vital genes is modified, ultimately affecting overall well-being [28,29,30]. Studies have shown that nutrients have an epigenetic impact on phenotypic and disease susceptibility throughout life. Folate, a water-soluble B vitamin, is a source of one carbon to produce S-Adenosyl methionine (AdoMet), which is required for DNA methylation; folate metabolism is connected to phenotypic alterations through DNA methylation [29,30,31]. Other methyl donor substances, including choline, can likewise change the DNA’s methylation status, which will then affect how genes are expressed [29]. A diet rich in methyl-donating nutrients can quickly affect gene expression, particularly in early development, when the epigenome is being established, and can have long term consequences in adulthood [32]. Animal studies have shown that an insufficient intake of folate or choline, which are methyl-donating compounds, before or after birth leads to lasting hypomethylation of certain genomic regions [33]. In adults, a diet lacking in a methyl group leads to reduced DNA methylation, but these changes can be reversed when methyl is reintroduced into the diet [32]. However, there is a gap in the literature in understanding the relationship between dietary intake of children and obesity-related gene methylation considering the racial disparities. In our previous study [34], we found that children who were overweight/obese (OW/OB) had increased methylation of the *NRF1* and *FTO* genes and decreased methylation of the *LEPR* gene when compared to normal weight (NW). Specifically, African American (AA) children had significantly higher methylation of *LEPR* compared to European American (EA) children. Along the line, this research aims to identify the dietary nutrients that are associated with the methylation of the obesity-related genes *NRF1*, *FTO*, and *LEPR* in racial disparities in childhood obesity.

## 2. Materials and Methods

### 2.1. Study Participants and Sample Analysis

Detailed information of the study participants has been previously given in Patel et al. [34]. Briefly, children aged 6 to 10 years were recruited from Lee and Macon counties, AL. A total of 113 children participated in this study. A phone survey was conducted with the parents to identify the children’s history of diabetes or cardiovascular disease to exclude them from the study. Children who self-identify as EA or AA ethnicity were included. Children were brought by their parents to Auburn University, and their anthropometric data and saliva samples were collected. The body weights and heights of the participants were measured based on the World Health Organization (WHO)’s guidelines. The children were classified as normal weight, overweight, or obese, based on the Centers for Disease Control and Prevention (CDC)’s standards [35]. Furthermore, saliva was used to isolate the DNA, which was then bisulfite converted for the MethyLight RT-PCR reaction. The multiplex PCR was carried out for the three genes *NRF1*, *FTO*, and *LEPR*, for which two primers and one probe for each gene were designed. Detailed protocols for each step have been mentioned in previous published article [34].

### 2.2. Dietary Nutrient Analysis

Parents were asked to complete a detailed 24 h dietary recall of the child, which consisted of two parts. First, they were asked to write down what the child consumed for breakfast, lunch, and dinner, as well as desserts, snacks, and drinks. The second part consisted of the consumption of major food groups including vegetables, fruits, bread/grains, oily fish, high-fat meat, lean meat, non-meat protein, eggs, milk and milk products, cheese, sugared beverages, sweets, potato or corn snacks, and caffeinated beverages, along with their serving sizes. Additionally, each group had sub-categories of specific food items. For instance, the vegetable group had two sub-categories, uncooked and cooked vegetables, which had serving sizes of 1 tennis ball and ½ a tennis ball, respectively. Similarly, for other groups, references were given for portion sizes such as 1 deck of cards, 1 golf ball, ounces, size of index finger, cubes, etc.

Using ESHA’s Food Processor Diet Analysis Software Version 11.11 (Salem, OR, USA), energy and nutrient intake were calculated. The Food Processor Diet Analysis program from the ESHA offered thorough reports on nutritional consumption, both macro and micronutrients. Over 1900 food sources, including the USDA Standard Reference database, USDA Food Data Central Brands, and manufacturer’s data, were included in the software database. Additionally, there were over 146,000 ingredients, recipes, and restaurant food brands in the software database.

### 2.3. Statistical Analysis

IBM SPSS Statistics 25.0 was used for all the statistical calculations. Based on the body weight, height, date of birth, and gender, the participants were divided into two groups: normal weight (NW) and overweight/obese (OW/OB). Similarly, participants were divided based on their racial groups: European American (EA) and African American (AA). To determine the differences in all the macro and micronutrient intake, an independent sample t-test was carried out between NW and OW/OB children as well as between EA and AA children. Pearson correlation coefficients were calculated to demonstrate the correlation between the DNA methylation of genes (*NRF1*, *FTO*, and *LEPR*) and each nutrient for the following categories: NW, OW/OB, EA, and AA. Additionally, the correlation was adjusted for variables, including maternal education, family income, gender, and age.

## 3. Results

The demographic details about the study population were given in the previous paper (1). Table 1 shows the PMRs of the *NRF1*, *FTO*, and *LEPR* genes and the nutrient intake by the children. In overweight/obese EA children, there was a notable rise in PMR (Percentage of Methylation Reference) for the *NRF1* and *FTO* genes, whereas no such increase was observed in the AA children. Conversely, the AA children had higher methylation levels of the *LEPR* gene among normal weight participants, but there were no differences in methylation between overweight/obese and normal weight EA children.

OW/OB children had a significantly higher intake of vitamin A-RAE (mg) (543.855 ± 39.448, *p* = 0.023) and retinol (mcg) (438.131 ± 33.648, *p* = 0.022) compared to NW children. There was also a significantly higher consumption of sodium (mg) (3618.817 ± 365.68, *p* = 0.03) amongst the OW/OB children compared to the NW, whereas the EA children had higher monosaccharide intake (4.511 ± 0.812, *p* = 0.002), vitamin A (IU) intake (4133.752 ± 856.042, *p* = 0.007), and vitamin C (mg) intake (185.838 ± 43.068, *p* = 0.04) compared to the AA children. On the other hand, the AA children had a significantly higher intake of vitamin B3 (mg) (18.804 ± 1.518, *p* = 0.005), vitamin B6 (mg) (1.628 ± 0.17, *p* = 0.011), vitamin B12 (mcg) (4.504 ± 0.49, *p* = 0.015), and Omega 6 (g) (9.07 ± 0.968, *p* = 0.012) than the EA children.

Furthermore, the differences between the dietary intake of normal weight EA and AA, and overweight/obese EA and AA children were identified using an independent sample *t*-test, shown in Table 2. AA normal weight children had significantly higher intake of PUFA (g) (11.337 ± 1.71, *p* = 0.028), added sugars (g) (50.736 ± 9.33, *p* = 0.036), vitamin B1 (mg) (1.29 ± 0.117, *p* = 0.014), vitamin B2 (mg) (1.709 ± 0.161, *p* = 0.017), vitamin B3 (mg) (19.549 ± 2.008, *p* = 0.001), iron (mg) (13.996 ± 1.148, *p* = 0.045), manganese (mg) (0.729 ± 0.115, *p* = 0.033), omega 3 (g) (0.886 ± 0.135, *p* = 0.042), and omega 6 (g) (9.652 ± 1.402, *p* = 0.021) compared to EA normal weight. But, the EA normal weight children had a higher monosaccharide (g) intake (4.625 ± 1.019, *p* = 0.012) than the AA normal weight children. Conversely, among the overweight/obese EA and AA children, the EA children had a significantly higher sugar intake (160.285 ± 18.053, *p* = 0.02) and vitamin A intake (4442.696 ± 1329.737, *p* = 0.048), whereas the AA children had a higher intake of vitamin B12 (mcg) (5.278 ± 0.852, *p* = 0.029).

Additionally, it was important to understand the correlation of the methylation of the *NRF1*, *FTO*, and *LEPR* genes with individual nutrients for all the four groups: NW, OW/OB, EA, and AA. Table 3 shows the Pearson correlation between the *NRF1* methylation and the nutrient intake. The correlation between the *NRF1* methylation and the NW children nutrient intake demonstrated a significantly positive moderate correlation with added sugar (r^2^ = 0.296, *p* = 0.022), oligosaccharides (r^2^ = 0.273, *p* = 0.035), vitamin B2 (r^2^ = 0.360, *p* = 0.005), vitamin B12 (r^2^ = 0.306, *p* = 0.017), folate (r^2^ = 0.363, *p* = 0.004), vitamin B6 (r^2^ = 0.385, *p* = 0.002), and iron (r^2^ = 0.352, *p* = 0.006). A stronger correlation was observed with Vitamin B1 (r^2^ = 0.455, *p* = 0.000) and vitamin B3 (r^2^ = 0.431, *p* = 0.001). On the other hand, a significant moderate correlation was observed between *NRF1* methylation and the OW/OB children’s trans-fat (r^2^ = 0.339, *p* = 0.013) intake and a stronger correlation with fluoride (r^2^ = 0.450, *p* = 0.001). In all EA children, a positively moderate correlation was observed with iron intake (r^2^ = 0.264, *p* = 0.037) and manganese (r^2^ = 0.358, *p* = 0.004), whereas it was soluble fiber (r^2^ = 0.288, *p* = 0.043) and pantothenic acid (r^2^ = 0.324, *p* = 0.022) for the AA children. A stronger correlation was seen with fluoride (r^2^ = 0.457, *p* = 0.001) in the AA children. Furthermore, to understand the role of family income, maternal education, race, and gender, the adjusted person correlation was calculated for the *NRF1* methylation (Table 4). The results showed a positive correlation with total fiber (r^2^ = 0.267, *p* = 0.046) intake in NW children after the adjusting, but the significance was lost for sugar, oligosaccharides, retinol, vitamin B2, vitamin B6, vitamin B12, folate, and manganese. If we look at the adjusted correlation in the OW/OB children, the significant correlation was lost for trans-fat. Among the races, the correlation was lost for iron in EA children.

Table 5 shows the Pearson correlation between *FTO* methylation and the nutrient intake. The correlation between *FTO* methylation and NW children nutrient intake demonstrated a moderate positive correlation with calorie intake (r^2^ = 0.258, *p* = 0.046), protein (r^2^ = 0.296, *p* = 0.022), carbohydrate (r^2^ = 0.286, *p* = 0.026), sugar (r^2^ = 0.264, 0.042), PUFA (r^2^ = 0.276, *p* = 0.032), vitamin B2 (r^2^ = 0.309, *p* = 0.016), iron (r^2^ = 0.303, *p* = 0.019), selenium (r^2^ = 0.298, *p* = 0.021), omega 3 (r^2^ = 0.279, *p* = 0.031), and omega 6 (r^2^ = 0.295, *p* = 0.022). While nutrients such as vitamin B1 (r^2^ = 0.492, *p* = 0.00) and vitamin B3 (r^2^ = 0.424, *p* = 0.001) demonstrated a stronger correlation. Similar to *NRF1*, the *FTO* methylation in OW/OB children for trans-fat (r^2^ = 0.316, *p* = 0.021) had a positive correlation. Along with that, disaccharide (r^2^ = 0.291, *p* = 0.035), fluoride (r^2^ = 0.295, *p* = 0.034), and manganese (r^2^ = 0.351, *p* = 0.010) were significantly correlated. In children who were EA, nutrients such as vitamin D (r^2^ = 0.277, *p* = 0.029), iron (r^2^ = 0.301, *p* = 0.017), and manganese (r^2^ = 0.482, *p* = 0.00) were positively associated. The intake of pantothenic acid (r^2^ = 0.332, *p* = 0.018) was the only nutrient that demonstrated a moderate positive correlation with *FTO* methylation in the AA children. When adjusted for family income, maternal education, gender, and age (Table 6), the significant correlations for calories, sugar, PUFA, vitamin B2, and omega 3 intake were lost. For OW/OB children, the significance was not seen in the trans-fat and disaccharide intake. After adjusting the variables, vitamin D intake in EA and pantothenic acid intake in AA children were not correlated with *FTO* methylation.

Table 7 shows the Pearson correlation between *LEPR* methylation and the nutrient intake. DNA methylation of the gene *LEPR* was moderately correlated with only one nutrient: manganese intake in NW children (r^2^ = 0.336, *p* = 0.009). OW/OB children’s intake of trans-fat (r^2^ = 0.361, *p* = 0.008) and fluoride (r^2^ = 0.477, *p* = 0.000) were strongly correlated. While, for EA children, added sugar (r^2^ = −0.253, *p* = 0.045), monosaccharide (r^2^ = −0.287, *p* = 0.023), and beta carotene (r^2^ = −0.312, *p* = 0.013) had moderate negative correlations. Similar to the OW/OB children, AA children’s intake of trans-fat intake (r^2^ = 0.294, *p* = 0.038) was correlated with *LEPR* methylation. After Pearson correlation, adjusting for family income, maternal education, race, and gender (Table 8), significance was lost for monosaccharide and added sugar among EA children and for trans-fat and pantothenic acid among AA children. This suggested that the relationship between methylation and the dietary nutrient intake may have been dependent upon the races, genders, family incomes, and maternal educations of the individuals.

**Figure 1:**
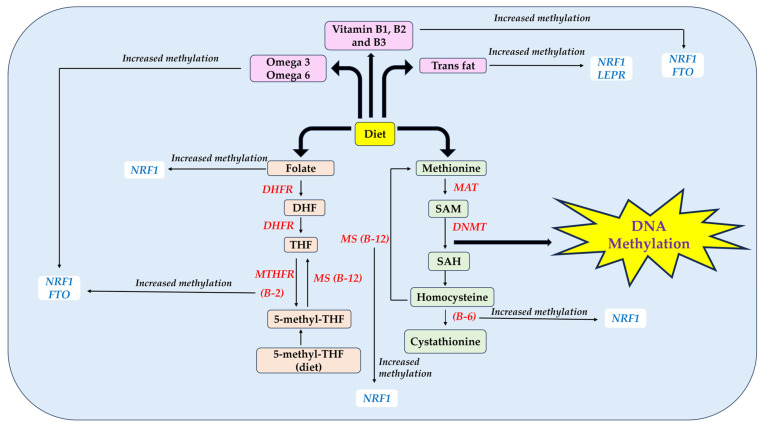
Summary of nutrients involved in one-carbon metabolism and the effects on the methylation of *NRF1*, *FTO*, and *LEPR* genes. B2, riboflavin; B3, niacin; B6, pyridoxine; B12, vitamin B12; B1, thiamine; NRF1, Nuclear Respiratory Factor 1; FTO, Fat mass and obesity associated; LEPR, Leptin Receptor; DHF, Dihydrofolic acid; THF, Tetrahydrofolate; SAM, S-adenosylmethionine; SAH, S-adenosylhomocysteine.

Lastly, Figure 1 shows the biochemical pathways involving various nutrients in the one carbon metabolism and the link towards the methylation of the *NRF1*, *FTO*, and *LEPR* genes.

## 4. Discussion

The study explored the association between the DNA methylation of the three genes *NRF1*, *FTO*, and *LEPR* with the nutrient intake of children. DNA methylation is a well-studied epigenetic modification that takes place in the one-carbon metabolism pathway. This process relies on specific enzymes and dietary micronutrients such as folate, choline, and betaine [36]. In this pathway, methionine is converted to S-adenosylmethionine (SAM), which serves as a methyl donor in cells. DNA methyltransferases (DNMTs) use SAM to attach methyl groups to the carbon-5 position of cytosine bases in DNA, resulting in the methylation of DNA [37].

Under normal conditions, folate that is consumed through a diet undergoes a process of metabolism in the intestine and/or liver, resulting in the formation of 5-methyltetrahydrofolate (5-methylTHF) in its monoglutamyl form, which further needs to be converted to tetrahydrofolate (THF) [38]. Polyglutamate synthetase is most effective when using THF as a substrate; thus, 5-methylTHF must be converted to THF through the methionine synthase reaction. Once THF is formed, either from folic acid or dietary folate, it is initially transformed into 5,10-methyleneTHF by the vitamin B6-dependent enzyme serine hydroxy methyltransferase. It is then irreversibly reduced to 5-methylTHF by methylenetetrahydrofolate reductase (MTHFR) [38]. This conversion is crucial for maintaining a steady supply of methyl groups used in the remethylation of homocysteine to methionine, which is facilitated by the vitamin B12-dependent enzyme methionine synthase [38]. Methionine serves as a substrate for SAM, a cofactor and methyl group donor involved in various methylation reactions, including the methylation of DNA, RNA, neurotransmitters, and other small molecules [39,40]. In this study, the dietary folate intake of NW children was associated with the DNA methylation of the *NRF1* gene. We have previously mentioned that the methylation level of *NRF1* in NW children was significantly lower than in OW/OB children [34]: the dietary intake of folate is lower in NW but not significantly different compared to OW/OB children. Ramos-Lopez and colleagues [41] conducted a cross-sectional study to examine how folate intake is related to the genomic methylation profile in a group of 47 obese participants from the Metabolic Syndrome Reduction in Navarra-Spain trial. They discovered that there were 51 CpGs (regions of DNA) that showed an association with folate intake. One of our genes of interest, *NRF1*, was associated with the innate immune response and plays a role in regulating various aspects of brown adipose tissue, including thermogenic adaptation, adipocyte inflammation, and cytokine production [42,43]. On the other hand, the gene *FTO* is responsible for controlling energy balance and eating behavior in specific regions of the hypothalamus, such as the arcuate, paraventricular, dorsomedial, and ventromedial nuclei [44]. Studies conducted in both living organisms and laboratory settings have shown that *FTO* can detect the nutritional status of the body and respond to appetite and food intake [45].

Several previous studies have examined the relationships between BMI, fat mass, and folate concentration in various age groups and populations [46,47,48,49]. One study found two possible explanations for the associations observed: firstly, obesity may lead to low serum folate levels, potentially affecting how the body processes folate and increasing the need for dietary folate. Secondly, low serum folate levels could be a contributing factor to obesity by affecting epigenetic modifications involved in lipid metabolism [50]. Another study supported these findings by showing that individuals with a BMI greater than 25 kg/m^2^ had lower serum folate levels, regardless of their folate intake [51]. This supported the hypothesis proposed by da Silva et al. that said that obesity may independently impact folate distribution by increasing the cellular uptake of dietary folate [52]. The findings of the above-mentioned studies could support our results of the OW/OB children having a higher methylation level for the *NRF1* gene, irrespective of their folate intake. It is interesting to note that the association was observed to be weaker after adjusting for the covariates of the study. Additionally, if we look at the methylation difference for *NRF1* in EA and AA normal weight children, there was a significant increase in the methylation of AA normal weight children, which could explain the positive association between *NRF1* methylation and folate intake. Furthermore, it would be interesting to see if the reduction in folate intake for OW/OB children could reduce the methylation of the genes.

Other methyl donors from the diet are vitamins B2 and B6. The riboflavin intake in the NW children of this study had a positive association with the methylation of the *NRF1* and *FTO* genes. In addition, the intake of riboflavin and the DNA methylation of the *NRF1* and *FTO* were significantly higher in OW/OB children. Similarly, significantly higher vitamin B6 intake and *NRF1* methylation were observed in AA children. Even though not significant, OW/OB children had a higher intake than the NW children. Previous research has shown that a higher intake of methyl donors could increase the methylation levels of the gene [53,54]. Even though not significantly associated with OW/OB children, normal-weight AA children had significantly higher vitamin B2 intake. Additionally, after adjusting for the covariates, the significant association was lost, indicating the impact of socio-economic status (maternal education, family income) gender, and age in the association.

The negative impacts of trans fatty acids in our diet and their effects on human health have been extensively studied and proven. Despite being restricted or prohibited in many countries, trans fatty acids might still lead to prolonged reactions that could raise valid concerns about human health, especially if they modify the epigenome [55]. Our study showed a positive correlation between *NRF1*, *FTO*, and *LEPR* methylation and OW/OB children’s trans-fat intake. Studies have been carried out to understand the role of fat intake on the epigenome [55,56,57,58], one of which suggested that the 54 genes associated exclusively with SFA CpGs have a significant impact on the liver’s metabolic functions, particularly in the regulation of glucose and insulin metabolism in obese adults [59]. Additionally, the study observed a higher correlation between DNA methylation of the CpGs associated with SFA and the presence of palmitic and stearic acid [59]. Unfortunately, we do not have human studies to support the association between trans-fat and DNA methylation.

Sufficient intake of micronutrients, including manganese (Mn), is crucial for proper fetal development. Imbalances in Mn levels, both deficiencies and excessive exposure, have been linked to the development of diseases later in life [60]. Bozack et al. examined the relationship between Mn levels in maternal erythrocytes during the first trimester and the presence of differentially methylated positions and regions in cord blood [60]. They also investigated whether these associations persisted in blood samples collected from children at mid-childhood (6–10 years old) in a cohort of 361 individuals. The study revealed that Mn levels were associated with increased methylation of a specific DNA site, cg02042823, located in the gene RNA binding fox-1 homolog 1 (RBFOX1 or A2BP1), in cord blood. This association remained significant but was weakened in blood samples collected during mid-childhood. The findings suggested a connection between prenatal levels of micronutrients, epigenetic modifications in the placenta, and birth weight [61]. The influence of Mn on epigenetic processes is an emerging field of research, and, thus far, only one human study has reported Mn-related changes in DNA methylation from birth to childhood. Our results showed increased methylations for all the three genes along with a significant correlation with the EA children manganese intake, suggesting to further conduct more race-specific human research for manganese and DNA methylation.

To our knowledge, this is the first study that demonstrated the association of the dietary intake of children with the methylation of obesity-related genes, considering the racial and health disparity effects. The prevalence rates of obesity varied significantly based on race and ethnicity. African Americans had a 50% higher likelihood of being obese compared to non-Hispanic whites [62]. These findings were consistent with another study that confirmed the higher risk of obesity among African Americans [63].

It has been observed that race, along with BMI status, is associated with the DNA methylation of genes, suggesting that epigenetic regulation may contribute to health disparities among different racial and ethnic groups [64]. Some significant changes were seen after adjusting the correlation in this study, implying the role of socioeconomic status in food availability, food security, and even the perception of healthy food. There is a growing body of evidence [65,66,67,68,69] supporting the connection between obesity and food insecurity, particularly for women, although the findings for children are still mixed. A study [70] revealed that both adults and children had high rates of overweight and obesity, and there was a significant prevalence of families that had recently experienced food insecurity. Living in a household that is food secure was linked to the perception that healthy food options are both affordable and convenient. Caregivers from food-insecure households experiencing hunger were found to have higher rates of unemployment and lower incomes compared to those from food-secure households [70]. Even though there were not many significant differences in the nutrient intake of OW/OB children, the lack of a significant correlation between nutrient intake and methylation of genes suggested the importance of environmental factors. The results also demonstrated the possible increased risk of normal-weight AA children becoming obese in the future. Along with this, it also opens the window to reevaluate the dietary needs for each nutrient based on their racial and socioeconomic status.

While our research established a connection between dietary nutrients and DNA methylation, as well as a potential association with health disparities, the limited size of our study sample might have hindered the identification of significant correlations with obesity indicators. To enhance the reliability and validity of the findings, it would be beneficial to broaden the scope of the current investigation and include larger sample sizes. Additionally, the diet intake of children was self-reported by the mothers, who could have under or overestimated certain portion sizes, affecting the overall dietary assessment.

## 5. Conclusions

To conclude, we found a significant association between the nutrient intake of children and the methylation of the *NRF1*, *FTO*, and *LEPR* genes. The study also highlighted the importance of health disparities in understanding the epigenetic effects of methyl donors in the diet and the methylation of genes. Furthermore, intervention studies can help observe the role of nutrient intake, specifically methyl donors in the diet in assessing the risk factors for childhood obesity.

## Figures and Tables

**Table 1:** General nutrient intake of children categorized by BMI and Race.

Nutrients	Normal Weight	Overweight/ Obese	*p* Value	European American	African American	*p* Value
PMR of *NRF1* (%)	68.925 ± 6.45	102.716 ± 13.031	**0.018**	75.692 ± 8.857	96.217 ± 11.592	0.155
PMR of *FTO* (%)	83.982 ± 17.499	168.24 ± 28.114	**0.010**	104.365 ± 19.412	147.612 ± 28.126	0.195
PMR of *LEPR* (%)	121.406 ± 13.544	85.078 ± 7.319	**0.025**	62.543 ± 2.307	157.065 ± 15.176	**0.000**
Calories (kcal)	2055.839 ± 110.41	2312.628 ± 118.573	0.116	2203.052 ± 113.764	2142.547 ± 116.239	0.714
Proteins (g)	75.416 ± 3.906	88.849 ± 6.974	0.086	83.291 ± 5.881	79.733 ± 4.852	0.653
Carbohydrates (g)	271.176 ± 16.539	303.724 ± 17.732	0.182	297.056 ± 17.906	273.068 ± 15.601	0.329
PUFA Fat (g)	9.187 ± 0.953	10.472 ± 1.356	0.432	8.861 ± 1.096	10.96 ± 1.198	0.200
Trans Fat (g)	0.214 ± 0.042	0.246 ± 0.056	0.647	0.216 ± 0.048	0.246 ± 0.049	0.657
Sugar (g)	109.305 ± 9.27	137.352 ± 12.289	0.067	130.186 ± 11.862	112.724 ± 8.678	0.259
Added Sugar (g)	38.791 ± 5.557	37.946 ± 7.282	0.926	34.928 ± 6.265	42.763 ± 6.403	0.389
Monosaccharide (g)	3.12 ± 0.63	3.122 ± 0.822	0.999	4.511 ± 0.812	1.37 ± 0.415	**0.002**
Disaccharide (g)	2.657 ± 0.558	2.867 ± 0.814	0.829	2.963 ± 0.513	2.493 ± 0.88	0.630
Oligosaccharides (g)	119.66 ± 6.833	158.091 ± 21.668	0.078	143.503 ± 18.634	130.355 ± 7.553	0.551
Vitamin A-IU (IU)	2723.929 ± 639.128	3116.007 ± 832.119	0.706	4133.752 ± 856.042	1363.155 ± 339.475	**0.007**
Vitamin A-RAE (mg)	424.487 ± 33.793	543.855 ± 39.448	**0.023**	479.89 ± 37.172	481.208 ± 36.895	0.980
Retinol (mcg)	334.447 ± 29.774	438.131 ± 33.648	**0.022**	350.436 ± 31.51	424.206 ± 32.143	0.108
Beta Carotene (mcg)	696.732 ± 130.705	830.128 ± 198.743	0.568	921.153 ± 178.052	555.361 ± 131.361	0.117
Vitamin B1 (mg)	1.095 ± 0.078	1.175 ± 0.101	0.527	1.043 ± 0.091	1.246 ± 0.082	0.108
Vitamin B2 (mg)	1.453 ± 0.105	1.634 ± 0.1	0.218	1.422 ± 0.092	1.685 ± 0.116	0.073
Vitamin B3 (mg)	15.682 ± 1.205	16.146 ± 1.437	0.804	13.595 ± 1.065	18.804 ± 1.518	**0.005**
Vitamin B3-NE (mg)	17.943 ± 1.267	17.947 ± 1.625	0.998	15.463 ± 1.075	21.07 ± 1.758	**0.005**
Vitamin B6 (mg)	1.315 ± 0.129	1.411 ± 0.141	0.613	1.147 ± 0.096	1.628 ± 0.17	**0.011**
Vitamin B12 (mcg)	3.336 ± 0.396	4.144 ± 0.424	0.166	3.088 ± 0.33	4.504 ± 0.49	**0.015**
Vitamin C (mg)	132.061 ± 32.341	151.565 ± 38.133	0.695	185.838 ± 43.068	84.976 ± 8.858	**0.042**
Vitamin D-IU (IU)	165.923 ± 17.027	216.319 ± 19.893	0.056	175.31 ± 17.498	207.703 ± 19.862	0.223
Folate (mcg)	267.393 ± 22.024	291.33 ± 30.814	0.522	263.297 ± 23.218	297.928 ± 30.041	0.356
Folate_DFE (mcg)	317.276 ± 35.577	373.59 ± 47.336	0.337	310.995 ± 35.098	384.883 ± 48.577	0.209
Pantothenic Acid (mg)	0.746 ± 0.092	0.691 ± 0.09	0.674	0.708 ± 0.091	0.736 ± 0.092	0.826
Fluoride (mg)	0.051 ± 0.034	0.012 ± 0.005	0.286	0.044 ± 0.033	0.019 ± 0.007	0.516
Iron (mg)	12.475 ± 0.739	14.885 ± 1.045	0.058	12.936 ± 0.861	14.449 ± 0.935	0.238
Manganese (mg)	0.566 ± 0.074	0.988 ± 0.485	0.364	0.867 ± 0.409	0.633 ± 0.087	0.615
Selenium (mcg)	45.071 ± 3.483	48.888 ± 5.108	0.530	44.638 ± 4.204	49.662 ± 4.311	0.411
Sodium (mg)	2799.205 ± 154.008	3618.817 ± 365.68	**0.033**	3291.305 ± 327.413	3047.947 ± 144.326	0.534
Omega 3 (g)	0.723 ± 0.078	0.745 ± 0.08	0.848	0.652 ± 0.066	0.835 ± 0.093	0.101
Omega 6 (g)	7.723 ± 0.815	7.214 ± 0.755	0.651	6.255 ± 0.609	9.07 ± 0.968	**0.012**

Data are expressed as mean ± SEM. *p* values are calculated with the *t*-test, which represents the statistical significance between NW and OW/OB participants and between EA and AA. Values in bold are statistically significant. *p* value was considered significant at 0.05 level.

**Table 2:** Differences between the dietary intake of normal weight and overweight/obese EA and AA children.

Nutrients	Normal Weight			Overweight/Obese		
	European American	African American	*p* Value	European American	African American	*p* Value
PMR of *NRF1* (%)	48.869 ± 4.254	90.364 ± 11.351	**0.001**	101.676 ± 15.738	104.3 ± 23.001	0.923
PMR of *FTO* (%)	28.955 ± 1.965	142.805 ± 33.036	**0.001**	177.419 ± 33.618	154.252 ± 50.007	0.691
PMR of *LEPR* (%)	64.074 ± 3.23	182.692 ± 22.981	**0.000**	61.061 ± 3.322	121.675 ± 14.647	**0.000**
PUFA Fat (g)	7.176 ± 0.793	11.337 ± 1.71	**0.028**	10.493 ± 1.991	10.44 ± 1.643	0.985
Sugar (g)	99.117 ± 13.414	120.197 ± 12.663	0.259	160.285 ± 18.053	102.406 ± 10.96	**0.020**
Added Sugar (g)	27.617 ± 5.74	50.736 ± 9.33	**0.036**	42.01 ± 10.972	31.752 ± 7.773	0.496
Monosaccharide (g)	4.625 ± 1.019	1.511 ± 0.604	**0.012**	4.399 ± 1.273	1.175 ± 0.542	0.054
Vitamin A-IU (IU)	3814.841 ± 1088.595	1557.781 ± 574.091	0.077	4442.696 ± 1329.737	1094.385 ± 169.807	**0.048**
Vitamin B1 (mg)	0.913 ± 0.094	1.29 ± 0.117	**0.014**	1.169 ± 0.152	1.186 ± 0.112	0.936
Vitamin B2 (mg)	1.215 ± 0.124	1.709 ± 0.161	**0.017**	1.623 ± 0.126	1.652 ± 0.168	0.886
Vitamin B3 (mg)	12.065 ± 1.051	19.549 ± 2.008	**0.001**	15.077 ± 1.814	17.775 ± 2.358	0.363
Vitamin B3-NE (mg)	14.42 ± 1.017	21.708 ± 2.199	**0.003**	16.474 ± 1.875	20.19 ± 2.935	0.268
Vitamin B12 (mcg)	2.766 ± 0.544	3.944 ± 0.566	0.139	3.4 ± 0.382	5.278 ± 0.852	**0.029**
Iron (mg)	11.052 ± 0.887	13.996 ± 1.148	**0.045**	14.76 ± 1.401	15.076 ± 1.589	0.884
Manganese (mg)	0.414 ± 0.089	0.729 ± 0.115	**0.033**	1.307 ± 0.799	0.5 ± 0.13	0.421
Omega 3 (g)	0.571 ± 0.074	0.886 ± 0.135	**0.042**	0.731 ± 0.107	0.765 ± 0.121	0.839
Omega 6 (g)	5.919 ± 0.769	9.652 ± 1.402	**0.021**	6.58 ± 0.949	8.227 ± 1.241	0.293

Data are expressed as mean ± SEM. *p* values are calculated with the *t*-test, which represents the statistical significance between NW and OW/OB participants and between EA and AA. Values in bold are statistically significant. *p* value was considered significant at 0.05 level.

**Table 3:** Pearson correlation coefficients (r^2^) for the relation between DNA methylation of *NRF1* gene and nutrients.

Nutrients	Normal Weight		Overweight/Obese		European American		African American	
	r^2^	*p* Value	r^2^	*p* Value	r^2^	*p* Value	r^2^	*p* Value
Trans Fat (g)	−0.080	0.543	0.339	**0.013**	0.221	0.082	0.178	0.216
Fiber Soluble (g)	−0.090	0.496	0.155	0.268	0.156	0.222	0.288	**0.043**
Added Sugar (g)	0.296	**0.022**	−0.252	0.069	−0.102	0.426	−0.063	0.665
Oligosaccharide (g)	0.273	**0.035**	0.050	0.723	0.197	0.122	−0.007	0.963
Retinol (mcg)	0.301	**0.020**	−0.083	0.554	0.113	0.379	0.041	0.777
Vitamin B1 (mg)	0.455	**0.000**	−0.067	0.636	0.032	0.802	0.183	0.204
Vitamin B2 (mg)	0.360	**0.005**	−0.045	0.749	0.079	0.537	0.143	0.321
Vitamin B3 (mg)	0.431	**0.001**	−0.120	0.393	0.094	0.464	−0.008	0.958
Vitamin B3-NE (mg)	0.421	**0.001**	−0.136	0.333	0.024	0.850	0.003	0.982
Vitamin B6 (mg)	0.385	**0.002**	−0.127	0.367	0.011	0.932	0.056	0.700
Vitamin B12 (mcg)	0.306	**0.017**	−0.150	0.283	0.020	0.877	0.016	0.915
Folate (mcg)	0.363	**0.004**	−0.069	0.622	0.048	0.710	0.083	0.569
Folate_DFE (mcg)	0.283	**0.029**	−0.011	0.935	0.125	0.329	0.051	0.727
Pantothenic Acid (mg)	0.188	0.150	0.231	0.096	0.074	0.563	0.324	**0.022**
Fluoride (mg)	0.015	0.907	0.450	**0.001**	−0.015	0.908	0.457	**0.001**
Iron (mg)	0.352	**0.006**	0.037	0.791	0.264	**0.037**	0.034	0.814
Manganese (mg)	0.261	**0.044**	0.240	0.084	0.358	**0.004**	0.039	0.787

Values in bold are statistically significant. *p* value was considered significant at 0.05 level.

**Table 4:** Adjusted Pearson correlation coefficients (r^2^) for the relation between DNA methylation of *NRF1* gene and nutrients.

Nutrients	Normal Weight		Overweight/Obese		European American		African American	
	r^2^	*p* Value	r^2^	*p* Value	r^2^	*p* Value	r^2^	*p* Value
Fiber Total (g)	0.267	**0.046**	−0.010	0.948	0.092	0.487	0.067	0.656
Fiber Soluble Total (g)	0.099	0.470	0.185	0.204	0.253	0.053	0.343	**0.020**
Fiber Soluble (g)	0.052	0.705	0.169	0.245	0.242	0.065	0.363	**0.013**
Vitamin B1 (mg)	0.347	**0.009**	−0.011	0.942	0.004	0.974	0.194	0.195
Vitamin B3 (mg)	0.305	**0.022**	−0.070	0.632	0.094	0.477	0.000	0.999
Vitamin B3-NE (mg)	0.306	**0.022**	−0.085	0.562	0.030	0.819	0.009	0.955
Folate (mcg)	0.295	**0.027**	0.026	0.860	0.042	0.751	0.098	0.516
Pantothenic Acid (mg)	0.189	0.163	0.233	0.107	0.127	0.338	0.321	**0.030**
Fluoride (mg)	0.064	0.638	0.439	**0.002**	−0.055	0.683	0.392	**0.007**
Iron (mg)	0.281	**0.036**	0.089	0.543	0.244	0.063	0.060	0.692
Manganese (mg)	0.234	0.082	0.237	0.102	0.359	**0.005**	0.043	0.775

*p* values are adjusted for maternal education, family income, gender, and age. Values in bold are statistically significant. *p* value was considered significant at 0.05 level.

**Table 5:** Pearson correlation coefficients (r^2^) for the relation between DNA methylation of *FTO* gene and nutrients.

Nutrients	Normal Weight		Overweight/Obese		European American		African American	
	r^2^	*p* Value	r^2^	*p* Value	r^2^	*p* Value	r^2^	*p* Value
Calories (kcal)	0.258	**0.046**	−0.189	0.175	0.057	0.658	0.027	0.851
Proteins (g)	0.296	**0.022**	−0.126	0.370	0.093	0.469	−0.005	0.974
Carbohydrates (g)	0.286	**0.026**	−0.232	0.094	−0.018	0.889	0.097	0.501
PUFA Fat (g)	0.276	**0.032**	−0.124	0.376	0.047	0.712	−0.001	0.993
Trans Fat (g)	−0.227	0.081	0.316	**0.021**	0.185	0.146	0.042	0.773
Sugar (g)	0.264	**0.042**	−0.170	0.222	0.052	0.685	0.050	0.730
Added Sugar (g)	0.323	**0.012**	−0.252	0.069	−0.095	0.461	0.006	0.969
Disaccharide (g)	−0.142	0.280	0.291	**0.035**	0.106	0.409	0.171	0.234
Vitamin B1 (mg)	0.492	**0.000**	−0.095	0.499	0.069	0.592	0.195	0.174
Vitamin B2 (mg)	0.309	**0.016**	−0.009	0.948	0.196	0.124	0.087	0.550
Vitamin B3 (mg)	0.424	**0.001**	−0.139	0.320	0.146	0.252	−0.008	0.954
Vitamin B3-NE (mg)	0.437	**0.000**	−0.181	0.195	0.064	0.618	−0.004	0.980
Vitamin D-IU (IU)	−0.015	0.911	0.035	0.801	0.277	**0.029**	−0.177	0.219
Pantothenic Acid (mg)	0.242	0.063	0.252	0.069	0.129	0.314	0.332	0.018
Fluoride (mg)	−0.043	0.742	0.295	**0.034**	−0.061	0.636	0.249	**0.081**
Iron (mg)	0.303	**0.019**	0.070	0.616	0.301	**0.017**	0.060	0.681
Manganese (mg)	−0.104	0.430	0.351	**0.010**	0.482	**0.000**	−0.156	0.278
Selenium (mcg)	0.298	**0.021**	−0.162	0.247	−0.002	0.988	0.013	0.928
Omega 3 (g)	0.279	**0.031**	−0.134	0.340	0.017	0.898	0.042	0.772
Omega 6 (g)	0.295	**0.022**	−0.063	0.659	0.084	0.511	0.040	0.782

Values in bold are statistically significant. *p* value was considered significant at 0.05 level.

**Table 6:** Adjusted Pearson correlation coefficients (r^2^) for the relation between DNA methylation of *FTO* gene and nutrients.

Nutrients	Normal Weight		Overweight/Obese		European American		African American	
	r^2^	*p* Value	r^2^	*p* Value	r^2^	*p* Value	r^2^	*p* Value
Proteins (g)	0.286	**0.033**	−0.112	0.443	0.064	0.629	0.013	0.933
Carbohydrates (g)	0.302	**0.024**	−0.108	0.461	0.041	0.756	0.093	0.539
Vitamin B1 (mg)	0.456	**0.000**	−0.053	0.718	0.041	0.755	0.233	0.120
Vitamin B3 (mg)	0.364	**0.006**	−0.098	0.504	0.141	0.286	0.006	0.971
Vitamin B3-NE (mg)	0.387	**0.003**	−0.137	0.349	0.066	0.621	0.002	0.991
Fluoride (mg)	−0.012	0.931	0.295	**0.042**	−0.116	0.387	0.211	0.159
Iron (mg)	0.248	0.066	0.170	0.244	0.284	**0.029**	0.128	0.395
Manganese (mg)	−0.155	0.255	0.336	**0.018**	0.495	**0.000**	−0.156	0.300
Selenium (mcg)	0.303	**0.023**	−0.193	0.183	−0.052	0.696	0.019	0.903
Omega 6 (g)	0.267	**0.046**	−0.118	0.425	0.036	0.787	0.018	0.908

*p* values are adjusted for maternal education, family income, gender, and age. Values in bold are statistically significant. *p* value was considered significant at 0.05 level.

**Table 7:** Pearson correlation coefficients (r^2^) for the relation between DNA methylation of *LEPR* gene and nutrients.

Nutrients	Normal Weight		Overweight/Obese		European American		African American	
	r^2^	*p* Value	r^2^	*p* Value	r^2^	*p* Value	r^2^	*p* Value
Trans Fat (g)	0.141	0.282	0.361	**0.008**	0.089	0.487	0.294	**0.038**
Added Sugar (g)	0.102	0.437	−0.229	0.099	−0.253	**0.045**	−0.050	0.732
Monosaccharide (g)	−0.121	0.357	−0.264	0.056	−0.287	**0.023**	0.151	0.295
Beta Carotene (mcg)	−0.173	0.186	−0.270	0.051	−0.312	**0.013**	−0.172	0.233
Pantothenic Acid (mg)	0.156	0.232	0.252	0.068	0.202	0.112	0.284	**0.046**
Fluoride (mg)	−0.047	0.723	0.477	**0.000**	0.241	0.059	0.017	0.908
Manganese (mg)	0.336	**0.009**	0.078	0.577	0.376	**0.002**	0.238	0.096

Values in bold are statistically significant. *p* value was considered significant at 0.05 level.

**Table 8:** Adjusted Pearson correlation coefficients (r^2^) for the relation between DNA methylation of *LEPR* gene and nutrients.

Nutrients	Normal Weight		Overweight/Obese		European American		African American	
	r^2^	*p* Value	r^2^	*p* Value	r^2^	*p* Value	r^2^	*p* Value
Trans Fat (g)	0.151	0.266	0.326	**0.022**	0.075	0.572	0.195	0.194
Beta Carotene (mcg)	−0.212	0.117	−0.247	0.088	−0.359	**0.005**	−0.173	0.250
Fluoride (mg)	−0.017	0.899	0.468	**0.001**	0.218	0.100	0.022	0.884
Manganese (mg)	0.296	**0.027**	0.082	0.575	0.371	**0.004**	0.206	0.170

*p* values are adjusted for maternal education, family income, gender, and age. Values in bold are statistically significant. *p* value was considered significant at 0.05 level.

## Data Availability

The study datasets of the current manuscript are available from the corresponding author upon request.
